# Management of Intraocular Pressure Elevation After Commercially Available Cultivated Oral Mucosal Epithelial Cell Transplantation for Limbal Stem Cell Deficiency

**DOI:** 10.7759/cureus.81341

**Published:** 2025-03-28

**Authors:** Hiroki Goto, Takashi Ono, Yukako Taketani, Mikiko Kimakura, Tetsuya Toyono, Takashi Miyai, Makoto Aihara

**Affiliations:** 1 Ophthalmology, University of Tokyo Hospital, Tokyo, JPN; 2 Ophthalmology, University of Tokyo, Tokyo, JPN

**Keywords:** corneal neovascularization, cultivated oral mucosal membrane transplantation, glaucoma, intraocular pressure, limbal stem cell deficiency, micropulse transscleral cyclophotocoagulation, regenerative medicine

## Abstract

Purpose: This study aimed to examine the changes in intraocular pressure (IOP) after commercially available cultivated oral mucosal epithelial cell transplantation (COMET) in Japanese patients with limbal stem cell deficiency (LSCD).

Methods: This retrospective observational study included consecutive patients who underwent commercially available COMET (Ocural®, Japan Tissue Engineering Co., Ltd. (J-TEC), Tokyo, Japan, and Nidek Co., Ltd., Gamagori, Japan) for LSCD at the University of Tokyo Hospital from January to December 2023. Their medical charts were reviewed for background information, IOP measurements, medication use, and surgery for IOP elevation before and one day, one week, one month, three months, and six months after COMET and the final follow-up point.

Results: Nine eyes from nine patients (mean age: 59.7±12.4 years) were included in the study. The primary diseases associated with LSCD were chemical burns (three eyes), Stevens-Johnson syndrome (two eyes), congenital aniridia (two eyes), and others (two eyes). IOP was significantly elevated from 9.7±4.1 mmHg before surgery to 23.0±12.3 mmHg one day after surgery (p=0.008) and to 20.2±7.0 mmHg one week after surgery (p=0.006). IOP was uncontrollable with eye drops in one eye (11.1%) two weeks after surgery, necessitating three sessions of micropulse transscleral cyclophotocoagulation. The mean number of antiglaucoma medications used did not change significantly during the observation period.

Conclusion: Commercially available COMET induces IOP elevation during the early postoperative period. Most cases of IOP elevation can be controlled with adequate management, but some instances of uncontrolled IOP necessitate careful consideration.

## Introduction

Limbal stem cell deficiency (LSCD) is a disease of the ocular surface caused by a disorder of the stem cells of the corneal epithelium located in the corneal limbus at the junction between the cornea and sclera [1]. Corneal limbal cells contribute to the optimal turnover of the corneal epithelium. When their function is defective, the corneal surface is invaded by the conjunctiva and neovascularization, leading to reduced corneal transparency and visual disturbance [2]. LSCD is caused by primary or secondary factors. Primary causes include aniridia, multiple endocrine deficiency, epidermal dysplasia, and dyskeratosis congenita [3]. Secondary causes include thermal or chemical burns, wearing contact lens, ocular inflammatory diseases, neurotrophic keratitis, and bullous keratopathy [4].

Treatment of LSCD is limited due to the difficulty in maintaining graft viability with conventional corneal transplantation. Therefore, cultivated oral mucosal epithelial cell transplantation (COMET) was developed to treat severe ocular surface disorders [5]. Autologous oral epithelial cells are cultured into sheets and transplanted to the eye with an amniotic membrane, yielding positive outcomes for severe ocular surface disease that otherwise has no effective treatment [5-10]. In recent years, COMET has become commercially available under the name Ocural® (Japan Tissue Engineering Co., Ltd. (J-TEC), Tokyo, Japan, and Nidek Co., Ltd., Gamagori, Japan) and has gradually become widely used in Japan. Postoperative intraocular pressure (IOP) elevation is a known adverse event following COMET, occurring in approximately 10% of reported cases [6]. Managing IOP after COMET is challenging due to the long duration required for the transplanted cells to cover the entire corneal surface, and antiglaucoma medications may delay re-epithelization. Knowledge about the frequency of IOP elevation, prognosis of elevated IOP, and appropriate treatments for elevated or uncontrolled IOP after Ocural® transplantation is limited. This highlights the importance of investigating the postoperative management of IOP. This study aimed to examine the changes in IOP after commercially available COMET in patients with LSCD.

## Materials and methods

This retrospective observational study was approved by the Ethics Committee of the Faculty of Medicine of the University of Tokyo Graduate School of Medicine (approval number: 2217-(15)) and adhered to the tenets of the Declaration of Helsinki. The need for written patient consent was waived due to the retrospective nature of the study, and informed consent was obtained from all participants through the opt-out method.

Patients

This study included patients who underwent commercially available COMET for LSCD at the University of Tokyo Hospital between January and December 2023. We retrospectively reviewed their medical records for age, sex, primary disease causing LSCD, IOP, central corneal thickness (CCT), and glaucoma history and treatment. Observation points were set at one day, one week, one month, three months, and six months post-surgery and the last day of follow-up. IOP was measured using a rebound tonometer (iCare Finland Oy, Vantaa, Finland), and the average of six measurements was used. The number of antiglaucoma medications was determined based on the reported mechanisms of action, with fixed drug combinations counted as two and oral acetazolamide as one [11]. CCT was measured using anterior segment optical coherence tomography (OCT) (CASIA2, Tomey, Japan).

Surgical procedure

Commercially available COMET was performed as previously reported [12], and the surgical technique is briefly presented below. The COMET is a two-stage procedure. After disinfection, a 5×17 mm section of the oral mucosa of the patients was collected and outsourced to a contractor (Japan Tissue Engineering Co., Ltd. (J-TEC), Tokyo, Japan; Nidek Co., Ltd., Gamagori, Japan) for culture. The intraoral wounds were sutured with three stitches using a 5-0 silk thread. The contractor treated the oral mucosal cells with enzymes, seeded them onto feeder cells, and cultured them into cell sheets. After approximately four weeks of culture, the oral mucosa cultured sheets were transported from the contractor and implanted on the ocular surface by ophthalmologists at the University of Tokyo Hospital. Following disinfection, the conjunctival tissue and blood vessels that invaded the cornea were bluntly detached and coagulated. After attaining adequate hemostasis, the oral mucosal sheet was sutured to the cornea with a 10-0 nylon thread, and the peripheral conjunctiva and sheet were sutured using an 8-0 absorbable thread. Finally, soft contact lenses were placed, and dexamethasone and ofloxacin ointments were applied. Postoperatively, 0.1% betamethasone and 0.3% gatifloxacin eye drops were administered six times daily and gradually tapered off. Postoperative IOP measurements were assessed after removing the soft contact lenses.

Statistical analysis

All values are described as mean±standard deviation. After testing for normality, the Friedman test with Dunn's multiple comparison tests was used to analyze IOP, CCT, and the number of antiglaucoma medications. Statistical analyses were performed using GraphPad Prism 9.5.1 (GraphPad Software, San Diego, California, United States). Statistical significance was set at p<0.05.

## Results

Nine eyes from nine patients (six males and three females) were included in the study. The mean age was 59.7±12.4 years (Table 1), and the mean follow-up duration was 252.4±105.1 days. The primary causes of LSCD were chemical burns in three eyes (33.3%), Stevens-Johnson syndrome in two eyes (22.2%), congenital aniridia in two eyes (22.2%), facial nerve palsy in one eye (11.1%), and chromosomal abnormalities in one eye (11.1%).

**Table 1:** Demographic background of patients who underwent commercialized cultivated oral mucosal epithelial cell transplantation for limbal stem cell deficiency

Patients' demographic background	
N (eyes)	9
Age (years)	59.7±12.4
Male/female (eyes)	6/3
Right/left (eyes)	5/4
Preoperative intraocular pressure (mmHg)	9.7±4.1
Preoperative central corneal thickness (μm)	555.3±161.3
Preoperative number of antiglaucoma medications	0.78±1.4

**Figure 1:**
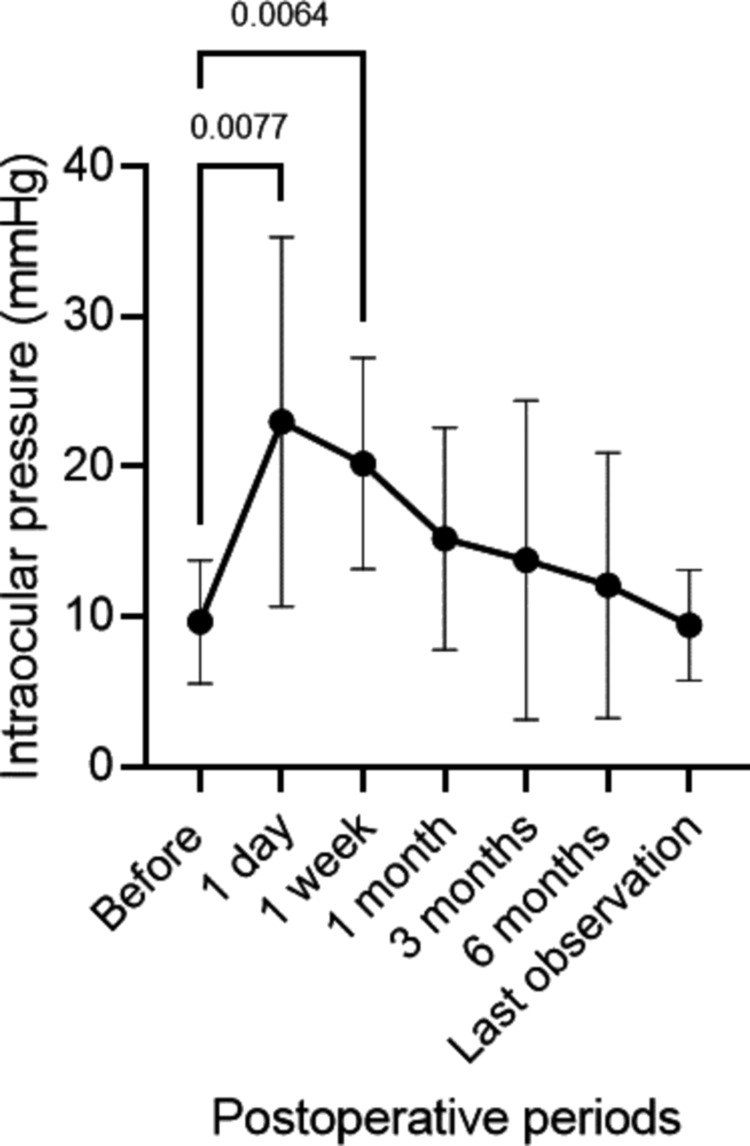
Changes in intraocular pressure after cultivated oral mucosal epithelial cell transplantation Intraocular pressure is significantly elevated from 9.7±4.1 mmHg before surgery to 23.0±12.3 mmHg one day after surgery (p=0.027) and 20.2±7.0 mmHg one week after surgery (p=0.006). No difference from the preoperative values was observed one month postoperatively.

**Figure 2:**
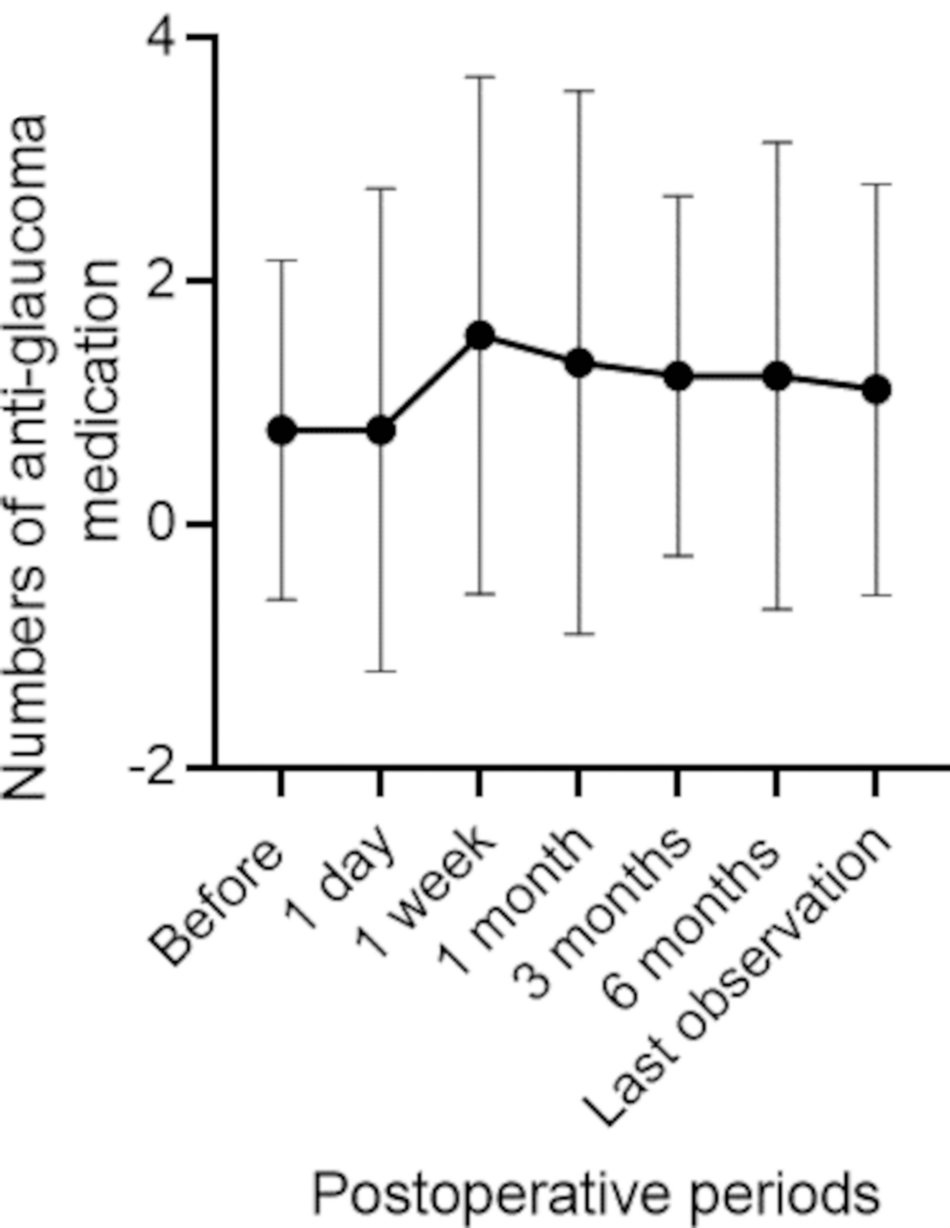
Perioperative transition of the number of antiglaucoma medications No significant differences in the number of antiglaucoma medications administered were observed after surgery.

**Figure 3:**
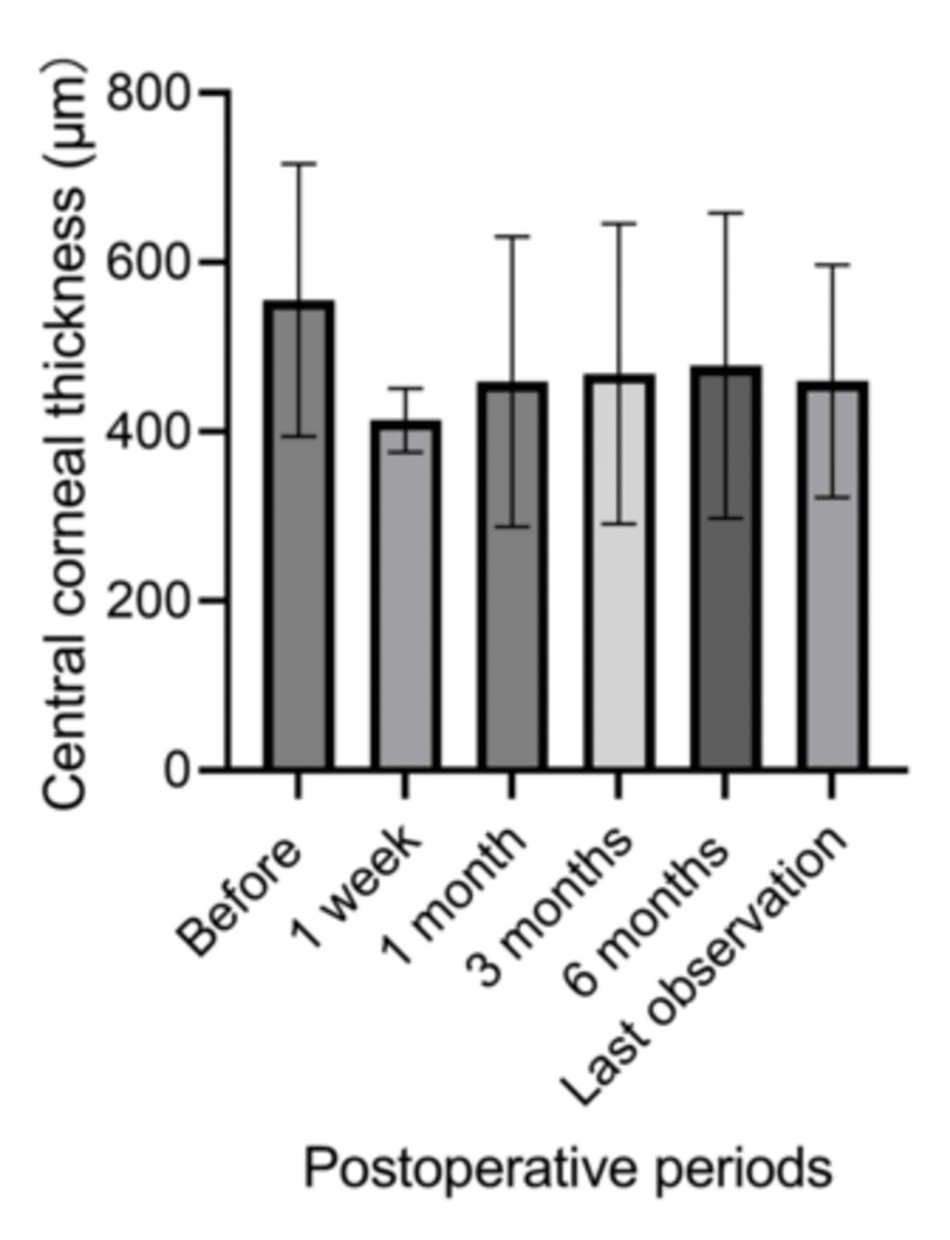
Changes in central corneal thickness after commercialized cultivated oral mucosal epithelial cell transplantation No significant difference in central corneal thickness was observed after surgery.

IOP significantly increased from 9.7±4.1 mmHg before surgery to 23.0±12.3 mmHg one day after surgery (p=0.008) and 20.2±7.0 mmHg one week after surgery (p=0.006) (Figure 1). The IOP returned to baseline levels, and no significant difference from the preoperative value was observed at one month postoperatively. The highest IOP postoperatively was observed after a mean of 10.7±13.1 days; the peak IOP was 29.6±12.0 mmHg. The number of antiglaucoma medications did not significantly change perioperatively (Figure 2). However, IOP remained uncontrollable in one eye (11.1%) at two weeks after surgery, presenting as more than 40 mmHg despite medication. This necessitated three sessions of micropulse transscleral cyclophotocoagulation (mpCPC). The other eight eyes (88.8%) did not require a surgical procedure to reduce the IOP. Table 2 summarizes the IOP changes and treatments. Overall, 77.8% (seven of nine eyes) experienced IOP elevation above 21 mmHg during the perioperative period. Perioperatively, 33.3% (three eyes) used antiglaucoma eye drops, while 66.6% (six eyes) required antiglaucoma drugs postoperatively (p=0.347). In contrast, CCT tended to decrease after surgery, although this change was not statistically significant for six months; the average values were <600 micrometers (Figure 3).

**Table 2:** Results and treatments for elevated intraocular pressure in patients who underwent commercialized COMET for limbal stem cell deficiency IOL: intraocular lens; mpCPC: micropulse transscleral cyclophotocoagulation; SJS: Stevens-Johnson syndrome; COMET: cultivated oral mucosal epithelial cell transplantation

Age	Sex	Primary disease	Preoperative antiglaucoma medications	History of glaucoma surgery	Lens state	Maximum postoperative intraocular pressure	Postoperative antiglaucoma medication at the final observation	Glaucoma surgery after COMET
74	M	SJS	0	-	Phakia	19	0	None
47	M	Chemical burn	0	-	IOL	22	0	None
55	M	Chemical burn	0	-	Phakia	25	1	None
44	M	Facial nerve palsy	0	-	Phakia	12	0	None
83	F	SJS	0	-	Aphakia	30	1	None
51	M	Chemical burn	0	-	Phakia	50	5	None
70	F	Congenital aniridia	4	Filtration device (Express) insertion/trabeculectomy/mpCPC	Aphakia	28	4	None
54	F	Chromosomal abnormality	2	-	IOL	37	4	None
59	M	Congenital aniridia	1	-	Aphakia	43	3	Three times of mpCPC

## Discussion

Our results demonstrate that IOP elevation occurred in 77.8% of the patients after commercially available COMET. While the COMET procedure does not involve intraocular manipulation, the poor corneal transparency in many cases makes it difficult to assess using slit-lamp microscopy. Nevertheless, postoperative inflammation in the anterior chamber of the eye is not typically intense. However, in this study, elevated IOP was observed in many cases, consistent with previous reports showing an incidence of 15-25% [13,14]. Therefore, the involvement of the episcleral vein is speculated to be the reason for IOP elevation. During COMET, adequate hemostasis of the blood vessels on the ocular surface must be ensured when removing the invaded conjunctival tissue from the cornea. This procedure can increase episcleral venous outflow resistance, leading to elevated IOP. Cauterizing the episcleral veins or laser photocoagulation has been used in experimental animal models of glaucoma [15,16]. Another possible cause of IOP elevation after COMET is the postoperative use of steroid eye drops [17]. We administered 0.1% betamethasone drops six times a day, which may have contributed to the steroid-induced IOP elevation. However, postoperative IOP elevation occurred in 27.3-28.6% of patients who used the same topical steroid regimen after keratoplasty, which required more invasive intraocular manipulation than COMET [18]. The ocular surface ablation and frequent use of steroid eye drops to prevent rejection are inevitable, and we need to anticipate the possibility of a transient increase in IOP sufficient to initiate IOP-lowering therapy promptly. The IOP elevation was temporary, but the IOP at the last observation was the same as the preoperative value after sufficient treatment in most cases. However, the IOP cannot be controlled using eye drops alone in some cases. Therefore, changes in IOP after commercially available COMET surgery should be considered. Furthermore, the IOP measured using iCare tended to be lower than that measured using applanation tonometry. Therefore, the actual IOP may have been higher than the values determined. Thus, the preoperative IOP of 9.7±4.1 mmHg may have been underestimated, and the postoperative value may have been higher. Therefore, caution is advised for changes in IOP, and they should be controlled with appropriate treatment.

IOP control is a major problem after COMET surgery because glaucoma eye drops can delay epithelial regeneration. The most common complications of COMET are epithelial defects (52.8%) and persistent epithelial defects (36.1%) [13]. For example, prostaglandin analog glaucoma eye drops are known to cause corneal epithelial damage [19,20], and carbonic anhydrase inhibitor eye drops cause histological changes in the corneal epithelium [21]. In addition to drug toxicity, patients may be allergic to various types of glaucoma eye drops, such as prostaglandin analogs, beta-blockers, carbonic anhydrase inhibitors, alpha agonists, and Rho-associated coiled-coil kinase (ROCK) inhibitors [22]. When multiple glaucoma eye drops are used, the total number of drops increases, and the side effects of preservatives such as benzalkonium chloride in the eye drops are likely to be more pronounced [23,24]. Unlike normal corneas, the epithelium takes several weeks or more to affix itself to the cultured sheets derived from the oral mucosa. During this period, a corneal epithelial defect continues forming on the cornea, leaving the epithelial cells in a fragile state. COMET aims to replenish epithelial stem cells on the ocular surface, and ophthalmologists may be hesitant to increase the use of eye drops that potentially harm these cells. Therefore, increases in antiglaucoma eye drops should be avoided in many cases, and other treatments, such as oral carbonic anhydrase inhibitors or combined medications, should be considered to reduce the number of eye drops. In our cases, the number of antiglaucoma eye drops did not significantly increase postoperatively due to concerns that epithelial regeneration would be inhibited. The increase in IOP is transient, but patients should be closely monitored to determine whether further postoperative eye drops are required for a prolonged period.

In addition to glaucoma eye drops, surgery is an option for reducing IOP after commercially available COMET. However, performing trabeculotomy can be challenging for many patients with LSCD because of insufficient corneal transparency, which makes intraocular surgery and preoperative angle evaluation difficult. Therefore, filtration devices and filtration surgery are considered alternative treatments. In one of our cases, the IOP was not lowered sufficiently, necessitating three sessions of mpCPC. Trabeculectomy is also difficult to perform if the conjunctiva is not adequately preserved to create a bleb as a bypass. Consequently, performing COMET is challenging in cases where the conjunctiva needs to be removed at 360°. In contrast, mpCPC is a newer IOP-lowering treatment option that has emerged in recent years and uses laser photocoagulation of the ciliary body to suppress aqueous humor production [25,26]. Unlike conventional cyclophotocoagulation, mpCPC can be performed at lower energy levels and is reportedly effective after keratoplasty [27]. With the anticipated increase in commercially available COMET procedures in the future, mpCPC is considered an effective treatment for managing high IOP, provided that careful observation of the ocular surface is maintained.

This study has several limitations. It had a retrospective design, and the sample was small due to the scarcity of ophthalmologic diseases requiring COMET. We plan to further investigate this issue on a larger scale in the future. The method for evaluating IOP was suboptimal. Applanation tonometry is commonly used to measure IOP. However, it is difficult to apply after COMET because of extremely large surface irregularities. Instead, we used the iCare IOP measurement system and averaged six measurements to derive more accurate values. Different rebound dynamics between normal corneal mucosa and oral mucosa-derived sheets were not analyzed. Therefore, the accuracy of IOP measurements using iCare for ocular patients requires further assessment. We anticipated changes in corneal thickness after ocular surface surgery, but significant differences were not found. There is a need to continue monitoring these statistics to determine if any significant differences exist with a larger patient cohort. The unstable state of the ocular surface causes difficulties when evaluating visual acuity or the visual field with increasing IOP. When the corneal epithelium has improved after COMET, penetrating keratoplasty should be applied followed by visual acuity and field assessments.

## Conclusions

Commercially available COMET induces IOP elevation during the early postoperative period, but this elevation can be controlled with adequate management in most cases. However, postoperative IOP fluctuations should be closely monitored in patients after COMET to prevent uncontrollably high IOP that may require multiple glaucoma surgeries.
